# Applicability of Metrology to Information Technology

**DOI:** 10.6028/jres.104.035

**Published:** 1999-12-01

**Authors:** Martha M. Gray

**Affiliations:** National Institute of Standards and Technology, Gaithersburg, MD 20899-0001

**Keywords:** function point metrics, information technology, software metrics

## Abstract

In 1959 the Director of the National Bureau of Standards declared “The emergence of science and technology as the paramount concern of the Nation in the 20^th^ century … demanded the highest order of measurement competence, in order to provide the standards and measurement techniques on which maintenance of scientific progress depended.” Since 1959, information technology has emerged as having a global impact on all facets of industry. However, the “standards and measurement techniques” needed to maintain the scientific progress of information technology into the next century may not be in place. This paper discusses the current state of software metrics.

## 1. Background

In *Measures for Progress: A History of the National Bureau of Standards*, Cochran describes the climate in 1900 when the legislation to create the National Bureau of Standards was proposed [1]. (The National Bureau of Standards became the National Institute of Standards and Technology, NIST in 1988.)

“The builders of America’s industrial complex had little interest in standards as such, but the scientists, engineers, and experimenters working for industry or independently found themselves increasingly hampered without them. The need for a Federal bureau of standards was talked about for almost 20 years before legislation for its establishment was introduced in 1900. By then the necessity had become imperative as science and industry, ready to take giant steps in the new century, looked for better measurements and more uniformity, precision, and control in the laboratory, factory, and plant.”

Metrology, the “science of measurement” [2], has advanced for some sciences such as chemistry and physics for over 200 years. Since information technology and computer science are fairly new sciences they have not been subjected to the metrological scrutiny that other sciences have. The importance of metrology has always been paramount to NIST. In 1959 the director, in a speech designed to generate support for new facilities, declared [3]:

“The emergence of science and technology as the paramount concern of the Nation in the 20^th^ century … demanded the highest order of measurement competence, in order to provide the standards and measurement techniques on which maintenance of scientific progress depended.”

This was the eve of the information age. The science of information technology and global use of computers were not even imagined. Yet the importance of measurement competence for scientific progress was deemed essential. We are still perhaps on the eve of “giant steps in the new century” for information technology. We will still need “better measurements and more uniformity, precision, and control” to achieve these “giant steps.”

## 2. Software Measurements

In most environments, measurement is an established, routine, customary part of daily practice. Many products are bought and sold based on what they weigh or what they measure. Measurement of software has not progressed to the point where there are established, routine, customary measures of software that are used in the daily development, purchase or sale of software. The oft quoted Lord Kelvin said [4]:

“When you can measure what you are speaking about and express it in numbers, you know something about it; but when you cannot measure, when you cannot express it in numbers, your knowledge is of a meager and unsatisfactory kind; it may be the beginning of knowledge, but you have scarcely, in your thoughts, advanced to the stage of science.”

What measurements are appropriate for information technology? If the field is narrowed, what measurements are appropriate for software? Grady and Caswell [5] state that “A software metric defines a standard way of measuring some attribute of the software development process. For example, size, cost, defects, communications, difficulty, and environment are all attributes. Examples of attributes in the physical world are mass, length, time, and the like.” This definition limits metrics to the software development process but it does connect the concept of metric to specific attributes of the software product. Some attributes, like size and time, certainly are easier to measure and more easily correlate to the physical world of metrology than defects or difficulty.

There are a large number of different types of metrics that are used for the software development process. Steve McConnell includes a table of “Useful Metrics” in his handbook on software construction that includes [6]:

**Table:** 

Size:	total lines of code written, total comment lines, total data declarations, total blank lines.
Productivity:	work-hours spent on the project, work-hours spent on each routine, number of times each routine changed, dollars spent on project, dollars spent per line of code, dollars spent per defect.
Defect Tracking:	severity of each defect, location of each defect, way in which each defect is corrected, person responsible for each defect, number of lines affected by each defect correction, work hours spent correcting each defect, average time required to find a defect, average time required to fix a defect, attempts made to correct each defect, number of new errors resulting from defect correction.
Overall Quality:	total number of defects, number of defects in each routine, average defects per thousand lines of code, mean time between failures, compiler-detected errors
Maintainability:	number of parameters passed to each routine, number of local variables used by each routine, number of routines called by each routine, number of decision points in each routine, control-flow complexity in each routine, lines of code in each routine, lines of comments in each routine, number of data declarations in each routine, number of blank lines in each routine, number of gotos in each routine, number of input/output statements in each routine.

Peng and Wallace [7] summarize metrics related to software error analysis in the following categories:

**Table:** 

Metrics for all phases:	problem metrics, cost and effort metrics, change metrics, fault metrics.
Requirements metrics:	primitive size metrics, requirements traceability, completeness, fault-days number and function points.
Design metrics:	size (primitive size metrics, number of modules), fault (primitive fault metrics), complexity (primitive complexity metrics, coupling, cohesion, structural fan-in/fan-out, information flow metric), design inspection (staff hours per major defect detected, defect density), test related (test related primitives).
Implementation metrics:	size (lines of code, halstead software science metrics), control structure (number of entries/exits per module, cyclomatic complexity), data structure (amount of data, live variables, variable scope, variable spans), test (primitive defect/error/fault metrics, fault density, defect age, defect response time, defect cost, defect removal efficiency, primitive test case metrics, statement coverage, branch coverage, path coverage, data flow coverage, test coverage), failure (mean time to failure, failure rate, cumulative failure profile).

McConnell’s table and Peng and Wallace’s list certainly suggest that there are a large number of metrics which can be used for software measurement. However, computer scientists and software engineers cannot usually even agree on what is important to measure, how to measure, or why we are measuring. Since the scientific process usually requires asking a question first, why are we trying to measure software” If we don’t know the why then the what and how are meaningless.

### 2.1 Why Measure

Since there are so many kinds of software measures there must be many reasons for measuring. As stated in the background section of this paper, NIST has traditionally stated that commerce, especially global commerce, is dependent on measurement technology so that buyers and sellers can agree on what is being bought or sold.

Roger Pressman states that [4]:

“Software is measured for many reasons: (1) to indicate the quality of the product, (2) to assess the productivity of the people who produce the product, (3) to assess the benefits (in terms of productivity and quality) derived from new software engineering methods and tools, (4) to form a baseline for estimation, and (5) to help justify requests for new tools or additional training.”

Stephen H. Kan states [8]:

“Measurement is becoming more important in software development. In this modern-day quality era, customers demand complex software solutions of high quality…. Furthermore, various software engineering techniques have emerged in the past decade: CASE tools, formal methods, software fault tolerance, object technology, and the like. To improve productivity and quality, software developers are faced with an enormous choice of methods, tools, and standards. However, as Fenton (1993) contended, there is very little quantitative data and objective evaluation of various methods in software engineering. There is an urgent need for proper measurements to quantify the benefits and costs of these competing technologies.”

Notice that both Pressman and Kan emphasize measuring software to improve quality and productivity, judge benefits, etc. Other authors mention reasons such as better understanding software development, controlling software projects, improving software development, estimating the costs or effort of a software development project, and facilitating the testing process.

### 2.2 Measure What

In traditional physical metrology theory there are established principals for quantities, units, scales, and uncertainty in measurement. A quantity is an “attribute of a phenomenon, body or substance that may be distinguished qualitatively and determined quantitatively” [9]. Examples include length, time, mass, and temperature. For these quantities, the concepts of units, scales and uncertainty are givens. If a desk is twice as long as a small table, the unit the desk is measured in is mathematically twice the units of the small table and the scale of this measurement would hold for other measurements. It also would be possible to identify the uncertainty of the measurement given the techniques used.

Information technology and more specifically software engineering have not traditionally used these concepts of fundamental units, scales, and uncertainty. There are volumes written on software measurements and metrics usually referencing measures for either the software product or the process of software development. The IEEE Standard Glossary of Software Engineering Terminology defines metric as [9]:

“A quantitative measure of the degree to which a system, component, or process possesses a given variable.”

This definition correlates to the definition of quantity given above from the *International Vocabulary of Basic and General Terms in Metrology* (VIM), but the concepts of units, scales and expressing uncertainty are not included [10]. They are also missing from most other definitions of metrics or measurements for software.

The IEEE Standard Glossary of Software Engineering Terminology also defines quality metric as [11]:

“(1) A quantitative measure of the degree to which an item possesses a given quality attribute. (2) A function whose inputs are software data and whose output is a single numerical value that can be interpreted as the degree to which the software possesses a given quality attribute.”

Peng and Wallace [12] differentiate the terms metric and measure defining metric as: “the mathematical definition, algorithm or function used to obtain a quantitative assessment of product or process.” They define measure as “the actual numerical value produced by a metric.”

Perhaps a more interesting definition is one that was eliminated from the 1990 edition (ANSI X3.172-1990) but included in the 1982 edition of the American National Dictionary for Information Processing Systems, ANSI X3/TR-1-82. This old edition contains a definition of “measure of information” [13]:

“(ISO) A suitable function of the probability of occurrence of an event or of a sequence of events from a set of possible events. In *information theory*, the term “event” is to be understood as used in the theory of probability. For instance, an event may be: — the presence of a given *element* of a set; — the occurrence of a specified *character* or of a specified word in a given *position* of a message.” (Italics are part of definition indicating words that are also defined in this dictionary.)

This definition is perhaps the most interesting because one of the problems in information technology and especially in software engineering has been the lack of metrics or measures that can be used to predict anything. Software is usually not delivered on time or under budget, sometimes being years late and factors of 10 off in the budget figures. Metrics on the uncertainty or predictability of the software’s behavior are not often used and remain almost impossible to determine.

### 2.3 Common Measures

When commercial off the shelf software (COTS) is purchased, the only measures listed on the labels are usually the hardware requirements for running the software package. For example, the software may say that 64 MB of RAM and 100 MB hard disk space are required to run the software. What the software delivers is not measured in any kind of units. For example, a word processing package does not say that this package delivers 20 units of some attribute. The package may list what functions are provided but there are no common units of measure for what is delivered or the functionality of what is delivered. The same may be said for any kind of commercial software package. Even if some common measure were determined, the 20 units that a word processing package delivers may not be as useful for the task at hand as the 20 units that a spreadsheet may deliver. A custom developed package may deliver 40 units to process your car insurance while a missile guidance system may deliver a very different 40 units.

This common measure is not only a problem for computer software. The most common measures for many things seem to be time and size. But for many complex things, size and time no longer seem to be the most important information for forming a judgement about the item in question.

For example, a tape of a movie at the video rental store tells how many minutes the movie runs in addition to the title, actors and actresses, etc. A person does not usually rent the tape because it has the most minutes anymore than they buy a software package because it is the biggest. The amount of minutes is important if a time limit is a problem but usually not the most important reason for a tape rental. A book is not usually judged by the number of pages but by the content of the pages. The functionality of the software and its ability to get the job done are what is important for software purchases, not usually the size of the software.

Another example of a common measure is time. Time is important for billing telephone calls but certainly does not indicate the content of the call. Runtime is important if the software is so slow that the user won’t use it or it can’t process the transactions required for the application. Certainly elapsed time is a critical factor in the development process and may partially determine if a product is going to be marketable. However the quality and functionality of the software may be more important than the time a product is delivered. Customers have been willing to wait for delayed products.

Thus time and size have some importance as measures but do not seem to be the most important for purchase decisions or usability of the software. Yet, size measurements and time measurements are the most common metrics used for software and application development, either as direct measures or derived measures as lines of code, productivity, costs, etc.

## 3. Size Measurements

### 3.1 Lines of Code

One of the most common forms of measurement for software development projects is lines of code (LOC) or thousands of lines of codes (KLOC), or standard lines of code (SLOC). Counts of lines of code are not an indication of the functionality or content of the program but they are an indication of the size of the program. Benefits of using LOC are that they are easily counted for all programs, there are models based on these measures, and much literature on these measures. From LOC other metrics can be developed such as productivity (LOC/staff-month) and quality (errors/LOC). If LOC is used for programs written in the same programming language, rates of errors, faults and failures can be compared. In addition, costs and documentation can be computed based on LOC. Problems with lines of code measures are that they can only be used after the product is completed, they are programming language-dependent, “they penalize well-designed shorter programs, that they cannot easily accommodate nonprocedural languages, and that their use in estimation requires a level of detail that may be difficult to achieve (i.e., the planner must estimate the LOC to be produced long before analysis and design have been completed.)” [14] Lines of code counts are especially difficult for estimating projects that involve new technologies. For example, how can LOC be used for a project that will involve client-server distribution using JAVA applets when local experience is with a mainframe COBOL application?

Another problem is that there are a variety of ways to count lines of code and controversy on what should be included, counting only executable lines, including data definitions, including comments, including job control commands. Thus knowing the number of lines of code does not allow you to make comparisons unless you know what types of lines were counted.

The other major problem with lines of code is described by T. Capers Jones [15]: “productivity measures expressed in source lines form paradoxically went backwards as real productivity improved…. The reason for this had been known for more than 200 years by manufacturing managers … if a manufacturing process involves a substantial percentage of fixed costs, and there is a decline in the number of units manufactured, then the cost per unit must go up. Software, as it turns out, involves a substantial percentage of fixed or inelastic costs that are not associated with coding. When more powerful programming languages are used, the result is to reduce the number of ‘units’ that must be produced for a given program or system, and the cost per unit must go up.”

### 3.2 Function Metrics

The Draft International Standard for the definition of functional size measurement [16] summarizes the need for a different sizing method in the introduction to the standard.

“Organizations engaged in software engineering have struggled for years in search of acceptable quantitative methods for measuring process efficiency and effectiveness, and for managing software costs, for the systems they acquire, develop, enhance or maintain. One critical, and particularly elusive, aspect of this measurement requirement has been the need to determine software size. Numerous software sizing methods have been proposed in the past. These included numbers of source lines of program code and various measures derived from the technical characteristics of the software.

These methods have limitations in that they can not be:

- applied early in the software development process,
- applied uniformly throughout the software’s life time,
- easily interpreted in business terms, or
- meaningfully understood by users of the software.

The concept of Functional Size Measurement (FSM) overcomes these limitations by shifting the focus away from measuring how the software is implemented to measuring size in terms of the functions required by the user.”

Alan Albrecht of IBM first publicized the function point metric in 1979. Function point metrics attempt to measure the size and complexity of the software not the number of lines of code that it takes to code the software. “Microsoft Word for Windows version 6.0, for example, has 5000 function points in total” [17]. Number of user inputs, number of user outputs, number of user inquiries, number of files and number of external interfaces are counted and weighed for complexity. Benefits of this metric include being programming language-independent (so they can be used for nonprocedural languages), and being easier to use as an estimating metric since most of the items that are measured are known before the coding begins. Function metrics have even been applied to object oriented analysis and design. “Opponents claim that the method requires some “sleight of hand” in that computation is based on subjective, rather than objective, data; that information domain information can be difficult to collect after-the-fact: and that FP has no direct physical meaning—it’s just a number” [18].

There have been some studies however, that seem to indicate that function points “are much more reliable than previously suspected” [19]. This was based on a study at the Massachusetts Institute of Technology. Another study completed at the University of New South Wales analyzed the “consistency and limitations of functions points as an *a priori* measure of system size compared to the traditional lines of code measure.” This study concluded that “function points are a more consistent a priori measure of system size” [20]. Function points are usually used like lines of code counts for productivity (FP/staff-month), quality (errors/FP), etc.

Function points “are derived using an empirical relationship based on countable measures of software’s information domain and subjective assessments of software complexity” [21]. “One of the initial design criteria for function points was to provide a mechanism that both software developers and users could utilize to define functional requirements…. herefore, one of the primary goals of FPA is to evaluate a systems’s capabilities from a user’s point of view…. From a user’s perspective, a system helps them do their job by providing five basic functions. Two of these address the data requirements of an end user and are referred to as data functions. The remaining three address the user’s need to access data and are referred to as transactional functions” [22]. These five basic functions are internal logical files, external interface files, external inputs, external outputs, and external inquiries.

Internal Logical Files (ILF) are “logical groupings of data in a system, maintained by an end user.” External interface files are “groupings of data from another system that are used only for reference purposes.” External inputs are those functions that allow users to add, change, and delete data in internal logical files. External outputs allow users to produce outputs of data that is either in internal logical files or external interface files. External inquiries allow users to select and display specific data from internal logical files or external interface files.

Once these basic counts are completed there are two adjustment factors used to calculate function points, the functional complexity of each function and a value adjustment factor. The complexity factors are low, average or high. The value adjustment factor is an attempt to weigh a system“s technical and operational characteristics. The scores from these fourteen adjustment areas may increase or decrease the unadjusted function point count. These fourteen areas have sometimes been called processing complexity factors or general application characteristics.

1. Data Communications—data and control information used in the application is sent or received over communication facilities including “various networks, concentrators, multiplexers, and private lines” [23] and LANs.
2. Distributed Data Processing—“the application uses data stored, accessed, or processed on a storage or processing system other than the one used in the main program routines” [24].
3. Performance—there are performance objectives such as response or throughput which influence the design, development, installations, and support of the application.
4. Heavily-used configuration—there are heavy use considerations or special main memory or storage considerations.
5. Transaction rate—there is a high transaction rate which influences the design, etc.
6. On line inquiry and data entry—these are characteristics which influence the security and control functions of the application.
7. End-User efficiency—there are human machine factors for “user-friendliness”, etc. which influence the design.
8. On-line update—there are requirements for on-line update of internal logical files. This effects the design of the system to include audit and recovery systems.
9. Complex Processing—there could be logical or mathematical complexities, control, security, or other attributes that add complexities.
10. Reusability—attributes which require analysis, planning, co-ordination or special design to interface with other programs or processing.
11. Conversion and installation ease—includes conversion and installation plan or tools.
12. Operational ease—includes purpose to “provide effective but easy startup, backup, error recovery, and shutdown procedures” [25].
13. Multiple sites—application designed, developed, and supported for multiple sites or implementations.
14. Flexibility—application has been designed, developed, and supported to facilitate change which includes planning for future maintenance and modification.

The concept of function points seems to be sound, i.e., that the size and complexity of software can be judged by items such as the number of files utilized. No matter what language was used to code such a system, the number of files utilized would remain the same.

## 4. Derived Measurements

Other measures are used for evaluating and estimating custom developed software. These measures are not usually used for commercial off the shelf packages. Measures exist for quality, complexity, reliability, and maintainability of the software, and productivity of the software developers. There are also measures for estimating the cost or the timing of a software development project. Quite often these measures use lines of code or function points as a basis for measures such as number of defects per lines of code, number of function points per staff month.

### 4.1 Quality Measures

Quality measures exist for the process of developing software and for the software product itself. Most quality measures relate to the software product itself but the increased acceptance of ISO 9000 standards has made some of the process model measures more widely used.

#### 4.1.1 Software Development Process Models

The development of software can be accomplished using many methods, processes, and models. Using the theory that better quality in the method, process or model used for development will produce better quality software, there is a growing body of measurements for evaluating or assessing the development process. Stephen H. Kan [26], describes software development process models and assessments which include:

1. The waterfall development model
2. The prototyping approach
3. The spiral model
4. The iterative development process model
5. The object-oriented development model
6. The clean room methodology
7. SEI Process Capability maturity Model (CMM)
8. SPR Assessment
9. Malcolm Baldrige Assessment
10. ISO 9000

These models and approaches define, evaluate, assess, etc. the way that software is developed, i.e., the development process itself. The measurements are of the process and the organization completing the process, not the final software that is produced. Most of the assessments have questions which have yes no answers such as was the version of the software clearly identified, were there page numbers in the documentation, was the correct approval identified, etc. At the end of this “audit” questions, such as, was the model or process closely or correctly followed, can be answered based on the number of positive answers.

These measures are measures of the software development process and can identify if the process or model was being followed but they do not assess the quality or attributes of the final software.

#### 4.1.2 Software Product Quality Measures

There are two types of software product quality measures: measures for errors, defects, faults, and failures and measures for more subjective judgements such as customer satisfaction. Errors, defects, faults, and failures usually use size measurements as part of the measures, e.g., number of defects per lines of code or number of defects per function point. User satisfaction is not as specific and may even be measured by number of calls to the help line or complaints to the support staff. All of these measures are used after a software product is developed, not before the development process.

The definitions for these errors, defects, faults, and failures vary widely. The IEEE Guide for the Use of IEEE Standard Dictionary of Measures to Produce Reliable Software [27], defines these terms as follows:

**Table:** 

Defect.	A product anomaly. Examples include such things as (1) omissions and imperfections found during early life cycle phases and (2) faults contained in software sufficiently mature for test or operation. See also “fault.”
Error.	Human action that results in software containing a fault. Examples include omission or misinterpretation of user requirements in a software specification, and incorrect translation or omission of a requirements in the design specification (see ANSI/IEEE Std 729-1983).
Failure.	(1) The termination of the ability of a functional unit to perform its required function. (ISO;ANSI/IEEE Std 729-1983). (2) An event in which a system or system component does not perform a required function within specified limits. A failure may be produced when a fault is encountered.
Fault.	(1) An accidental condition that causes a functional unit to fail to perform its required function. (ISO;ANSI/IEEE Std 729-1983). (2) A manifestation of an error in software. A fault, if encountered, may cause a failure. Synonymous with bug.

There are a number of metrics that relate the number of problems, errors, faults, or defects (e.g., defect density metric) per lines of code of function points. Sometimes these are tracked per release or per phase in the development process. These metrics usually include the number of problems identified, when they were corrected, what type of problem (e.g., orthogonal defect classification), and probable cause (defect cause analysis).

#### 4.1.3 Complexity Metrics

There are metrics that are used to describe the actual software itself, the design and coding structures of the software. These metrics can be used on traditional software, after the software is written to determine how complex the software is and perhaps indicate what the rate of defects might be. Additional studies of the correlations between complexity and defects haven’t proven the correctness of these metrics but work is ongoing for some of them.

##### 4.1.3.1 Halstead’s Measures for Software Science

Halstead proposed, in 1977, that a computer program is a “collection of tokens that can be classified as either operators or operands” [28]. Based on the number of distinct operators and operands that appear in a program, Halstead developed equations for estimating the length of the program, number of faults, complexity, and other factors.

This software science has had its criticism and later studies have not confirmed the validity of the equations. This work did, however, stimulate other research in software science and metrics.

##### 4.1.3.2 McCabe’s Cyclomatic Complexity

McCabe proposed in 1976 that a measure, cyclomatic complexity, could be developed based on the number and kind of independent paths in a program, that could be used to indicate a program’s testability and maintainability. Many studies have been completed on the relationship of this metric based on decisions and branches and defect rates of the software. Some suggest strong correlations for complexity and defects while other suggest that this correlation lessens when adjusted for program size.

##### 4.1.3.3 Syntactic and Structure Metrics

There are other measures that have been used which measure either the syntax of the software, for example whether loops are used or IF-THEN-ELSE statements, or the structure of the software, for example, the number of modules used, the number of modules called. These software design metrics seem to be used the same way that the cyclomatic complexity metrics are used, to help judge the quality of the software.

All of these quality metrics have one thing in common. They can only be used to judge the software after the software is written. Their use may suggest better design techniques and techniques which may improve the quality of the software, but they are not useful for estimating the size or cost of a software development project.

### 4.2 Estimation

One of the most plaguing problems in the software industry is the problem of estimating the schedule and cost of software development projects. Historically projects have been typically described as over budget and delivered late. “52.7 % of projects were over budget and late with fewer functions than specified” [29]. When new development techniques, new development languages, or new application platforms are introduced most estimation models have not been very accurate. The highest accuracy rate occurs when there is a good historical baseline available for comparison.

The Software Engineering Laboratory (SEL) of the Institute for Information Technology of the National Research Council of Canada published a report, *Software Cost Estimation and Control* [30] based on their study of software cost estimation methodologies. They classified the estimation process as either model based or analogy based. They describe formal models as follows:

“Formal models attempt to quantify all input to the cost estimation process, and then apply a set of equations that describe the relationships between the inputs and the outputs of the cost estimation process. The equations are developed through analysis of historical data and must be calibrated to each individual development environment.”

The Software Engineering Laboratory found that most organizations did not use formal methods. There were two reasons cited for not using these models [31]. “First, there was a lack of confidence in the ability of a model to outperform an expert. Managers felt that these models were expensive to implement and provided little benefit…. The second problem with the models is the lack of historical data available to calibrate the model. Proponents of models emphasize the fact that models are not transferable between organizations and that there is a great deal of effort required to calibrate a model for a particular organization. Without calibration, values produced by the model can fluctuate radically.”

#### 4.2.1 COCOMO

In 1981 Barry Boehm introduced COCOMO (COnstructive COst MOdel). COCOMO is software estimation models for custom designed software. The basic model can be used to estimate a software development effort and cost based on program size given in estimated lines of code. The intermediate model computes the software development effort based on program size and a set of other attributes that include characteristics of hardware, personnel, and the software development process. The advanced model includes all of the variables of the intermediate model plus adds the impact of these other attributes on each step of the development process.

There are three classes of software projects which have COCOMO models defined; small, simple software projects, medium software projects with a mix of requirements, and embedded software. The results of using these popular models have not been stellar. Estimates seem close to the actual completed project only when the historical data used for comparison is very close to the variables of the estimated project, that is, when the types of software, language used, and experience of the programmers are similar. This has not proven to be very successful when new technologies are introduced.

#### 4.2.2 Putnam Estimation Model

The Putnam Estimation Model relates number of lines of code to effort and development time. It assumes a specific distribution of effort over the life of a traditional software development project (definition, functional design and specification, design and coding, testing, operation and maintenance). This model is usually used to show how additional effort in one phase of the development cycle can shift the delivery schedule.

#### 4.2.3 Esterling Time-Study Model

The Esterling model is a more in-depth study of the interaction of programmers and the environment of the software development project. Esterling analyzed the amount of productive work from staff after considering nonwork interruptions, interruptions from staff working on projects, administrative distractions, and overtime. He then graphed the relative project cost, estimating scenarios for “best case” programmers, “typical” programmers, and “worst case” programmers. Based on this research, Esterling made the case that adding addition staff to programming projects may actually cost more money and slow development time compared to just leaving the existing number of staff members.

#### 4.2.4 Estimation by Analogy

The Software Engineering Laboratory (SEL) of the Institute for Information Technology of the National Research Council of Canada issued report on software cost estimation which stated that “The bulk of the current literature and research on cost estimation is devoted to formal models, particularly as relates to new system development….we found that formal models are not in general used by estimators as a primary tool for cost estimating” [32]. “By an overwhelming majority, informal analogy was the most commonly used estimating method for all types of software and for all organizations. Estimators used their past projects as a basis for estimating the cost of future projects” [33].

Informal analogy based estimating involves comparing a past project with the current project. Most often there is no database of appropriate data for the comparison but simply the memory of past participants. “The database consisted of their memories or the memories of their colleagues. Estimators often admitted that some information was available on past projects, but it was either in a form too difficult to access, or they did not believe accessing the information would improve the accuracy of the estimate” [33].

If there is data available, the SEL found that the data usually was not accurate, was not accessible, or not specified in a useful way. As an example, time sheets may be the only data source for time and labor breakdowns. A cost center does not always indicate what project or part of a project was actually being worked on. The data from time sheets may also only be in paper form since the personnel files may not be accessible.

The SEL found that the only time fairly accurate cost estimates of software projects were achieved was in situations with the following characteristics [34]:

- The users are experienced in the system, know what they want, and can express what they want.
- The requirements are clear, precise, correct, and complete.
- The project duration is short.
- The manpower loading is small.
- The people doing the estimation are experienced in the application domain and have developed similar systems.
- The development environment and development process are familiar to all people involved.
- Staff turnover is low both among the developers and the users.
- No unfamiliar software or hardware from outside suppliers is to be integrated with the final product.”

#### 4.2.5 Estimation Using Function Points

Capers Jones [35] states that, “One of the major challenges of software cost and schedule estimation is “sizing,” or predicting the amount of source code and other deliverables that must be built to satisfy the requirements of a software application. Sizing is a critical precursor to software cost estimating, whether estimation is done manually or by means of a commercial software cost estimating tool.” Function points can be used to size a software development project. They cannot be used directly to estimate costs, effort, or delivery time because variables such as the experience of the staff, tools used, and methodology used for development also affect costs, effort and delivery time. Past experience or industry benchmarks can facilitate these estimates. There are also other tools based on function points which estimate paper deliverables such as documentation and test cases for the software project.

If there is not a historical database of past projects, personnel and organizations involved, that is comparable to a new project, formal models for estimating are difficult to use. On the other hand, estimation by analogy has not proven to be very accurate. Using function points for estimation seems promising both for formal model use and for estimating projects using new technologies.

The International Function Point Users Group has supported efforts to validate function point counts and to establish industry benchmark data for function points. “One form of that information which has become increasingly popular is industry benchmark data. Benchmark data includes data used to quantify individual project performance levels and to provide information on the productivity and quality impact of various tools, techniques, and methods. The good news is that the software industry is mature enough to use valid benchmark data. It is also immature enough, or evolutionary enough, to have insufficient data available, in some cases, for reasonable statistical analysis” [29]. Major problems are that the data is not always current, does not include newer technologies or tools, and is not always consistent.

Estimation is used not only for complete projects. One current problem, for example, is the year 2000 problem. “Is counting lines of code a good way to estimate your workload for year 2000 conversion” Though that’s the most common method used, it’s not necessarily the best…. Not all languages have an accurate definition of what a line of code is. That includes Query by Example, Visual Basic, many database languages, and spreadsheets such as Lotus and Excel …” [17]. Capers Jones [17] believes function points can be used for this estimation problem. “For example, each reference to a calendar date in an application requires approximately one function point to encode.”

Roger Heller summarizes his views of function points as [36]:

“In conclusion, Function Point Analysis has proven to be an accurate technique to size, document, and communicate a system’s capabilities. It has been successfully used to evaluate the functionality of real-time and embedded code systems such as robot-based warehouses and avionics as well as traditional data processing. As computing environments become increasingly complex, it is proving to be a valuable tool that accurately reflects the systems we deliver and maintain.”

Capers Jones [37,38] also suggests that function points are now being used for “software quality studies, software contract management, business process re-engineering (BPR), and software portfolio control. The Internal Revenue Service is also exploring the usage of function point metrics for software taxation purposes.” Thus the use of function point metrics seems to be expanding. Perhaps the great danger with function points is the misuse of the metrics. Function points are only a size measurement.

“Function Points are a measure of software size based on an evaluation of the logical user requirements. Similar to the square feet of a house, function points are independent of the development methodology, tools or language used to build the software…. Just as the square-foot size of a house does NOT equal the speed at which a house can be built or its construction time, the Function Point size does NOT equal productivity or work effort.”

Function points are supported by the International Function Point Users Group (IFPUG). There is an IEEE Standard [39] and a proposed international standard for function points. IFPUG has created an established counting practices manual and supports committees developing case studies and approaches for new technologies. Function points seem to be the only appropriate software measurement available now for large complex, diverse projects [40, 41].

## 5. Summary

If future progress is dependent on better measurements and measurement techniques then perhaps we need a new perspective on software measurement. Although this paper is not an exhaustive survey of all software measurements activities, there seems to be no identified basis for software measurement which can apply the principals of physical metrology. Just as we count bits for the capacity of disks, etc. the basis for most of the software measurements used today is some kind of size measurement which is then combined with other measures. Both size measurements, lines of code (LOC) and function points, have problems with the principals of units, scale and measuring uncertainty.

There is very little published about fundamental measures for information technology or for computer software, although some discussion has begun to surface [42]. There is almost nothing relating these measures to other measurement concepts such as uncertainty and traceability. Capers Jones [43] believes that “… this basic problem of measurement is one of the biggest obstacles now facing the software industry.” Since business and modern society has become dependent on information technology both for a competitive edge and a necessary tool, it seems appropriate that further research is needed in this area.
